# Polyphenol-based polymer nanoparticles for inhibiting amyloid protein aggregation: recent advances and perspectives

**DOI:** 10.3389/fnut.2024.1408620

**Published:** 2024-07-29

**Authors:** Shuzhen Fang, Kangyi Zhang, Danqing Liu, Yulong Yang, Hu Xi, Wenting Xie, Ke Diao, Zhihong Rao, Dongxu Wang, Wenming Yang

**Affiliations:** ^1^The First Affiliated Hospital of Anhui University of Chinese Medicine, Hefei, Anhui, China; ^2^Center for Xin'an Medicine and Modernization of Traditional Chinese Medicine, Institute of Health and Medicine, Hefei Comprehensive National Science Center, Hefei, Anhui, China; ^3^Key Laboratory of Xin'an Medicine, Ministry of Education, Hefei, Anhui, China; ^4^State Key Laboratory of Tea Plant Biology and Utilization, Key Laboratory of Food Nutrition and Safety, School of Tea, Food Science and Technology, Anhui Agricultural University, Hefei, China; ^5^National Resource Center for Chinese Materia Medica, China Academy of Chinese Medical Sciences, Beijing, China; ^6^School of Grain Science and Technology, Jiangsu University of Science and Technology, Zhenjiang, China

**Keywords:** amyloidogenic property, polyphenols, nanoparticles, brain targeting, nanomedicine

## Abstract

Polyphenols are a group of naturally occurring compounds that possess a range of biological properties capable of potentially mitigating or preventing the progression of age-related cognitive decline and Alzheimer’s disease (AD). AD is a chronic neurodegenerative disease known as one of the fast-growing diseases, especially in the elderly population. Moreover, as the primary etiology of dementia, it poses challenges for both familial and societal structures, while also imposing a significant economic strain. There is currently no pharmacological intervention that has demonstrated efficacy in treating AD. While polyphenols have exhibited potential in inhibiting the pathological hallmarks of AD, their limited bioavailability poses a significant challenge in their therapeutic application. Furthermore, in order to address the therapeutic constraints, several polymer nanoparticles are being explored as improved therapeutic delivery systems to optimize the pharmacokinetic characteristics of polyphenols. Polymer nanoparticles have demonstrated advantageous characteristics in facilitating the delivery of polyphenols across the blood–brain barrier, resulting in their efficient distribution within the brain. This review focuses on amyloid-related diseases and the role of polyphenols in them, in addition to discussing the anti-amyloid effects and applications of polyphenol-based polymer nanoparticles.

## 1 Introduction

Between the years 2000 and 2020, there was a decrease in deaths attributed to stroke, heart disease, and human immunodeficiency virus, whereas reported deaths from Alzheimer’s disease (AD) saw a notable increase of over 145% (1). The incidence of deaths attributed to AD was further exacerbated in 2020 due to the global COVID-19 pandemic. Concurrently, the COVID-19 poses significant challenges and intricacies for caregivers of individuals with dementia. During the past two decades, the primary therapeutic approaches for AD have encompassed interventions targeting anti-neurotoxic *β*-amyloid (Aβ) aggregates, anti-Tau, anti-neuroinflammatory, neuroprotective agents, and brain stimulation, among others (2–4). The accumulation and deposition of Aβ, particularly Aβ_42_, serve as the primary and initiating etiological factor in the pathogenesis of AD (5, 6). Consequently, investigations involving Aβ_42_ aggregates in both *in vivo* and *in vitro* settings have consistently formed the cornerstone of research in this area. Due to the metastability and heterogeneity of Aβ (mainly Aβ_42_) aggregates, the pathogenesis of Aβ is inevitably diverse and complex (7, 8).

In recent years, there has been significant advancement in the field of nanotechnology, particularly in the development of emerging nanomaterials for the construction of nanoparticle-based drug delivery systems. This has garnered increasing attention in the fields of medicine and biology for their potential applications in disease diagnosis and treatment (9, 10). Nanoparticles exhibit a dense structure, enabling the attachment of drugs through mechanisms such as adsorption, dissolution, encapsulation, or covalent bonding (11–13). Nanoparticles have the capability to stabilize labile pharmaceutical compounds, enhance their aqueous solubility, extend the duration of drug or contrast agent presence in the circulatory system, and mitigate the inherent shortcomings of pharmaceutical compounds (14, 15). Furthermore, specialized targeting molecules can be employed to modify the nanoparticles for enhanced penetration of the blood–brain barrier, allowing for targeted action on specific cells or intracellular compartments (16, 17). Therefore, nanoparticles show great potential for enhancing pharmaceutical compounds delivery to specific central nervous system targets and facilitating pharmaceutical compounds release at sites of disease.

Polyphenols are commonly present in natural plant sources and exhibit diverse biological properties, particularly in the mitigation of AD and other neurodegenerative conditions (18, 19); however, their absorption and utilization within the human body are limited (20, 21). Hence, it is imperative to develop a strategy for harnessing the potential of polyphenols within the organism. This review provides an overview of the characteristics of amyloid and associated diseases, the inhibitory properties of natural polyphenols on amyloid fibrils, and the therapeutic effects and potential applications of polyphenol-based polymer nanoparticles.

## 2 Amyloidogenic property and diseases

Proteins are fundamental biomolecules that constitute the building blocks of life, serving as essential components for executing biological functions within organisms and contributing to various physiological processes. The preservation of proper protein structure is essential for the normal physiological functioning of proteins. Any deviation from the correct structure of a protein molecule is likely to result in the impairment of its function and the onset of organic pathology (22). Amyloid refers to a series of fibrillar proteins with abnormal protein conformation due to misfolding, which tend to aggregate with each other and form insoluble amyloid fibrils that are deposited in the body, thus triggering diseases, i.e., clinically recognized protein conformation diseases (23, 24). Currently, there exist over 20 types of protein conformational diseases, such as the association of Type 2 diabetes (T2D) with pancreatic islet amyloid polypeptide; AD with Tau protein and β-amyloid protein (25, 26); Parkinson’s disease with α-nucleosynthetic protein (27, 28); and transmissible spongiform encephalopathy with prion protein; Huntington’s syndrome with polyglutamine aggregation, among others (29). The amyloid proteins associated with each of these diseases have fibrillar properties in a β-folded structural conformation and are deposited intracellularly or extracellularly to form amyloid plaques (30).

The investigation of these amyloid-related diseases has emerged as a prominent area of pharmacological research. These type of disease share the following common characteristics: (1) while the sequences of individual proteins may vary, the resulting aggregated properties exhibit a high degree of similarity, characterized by a structured and insoluble nature; (2) the formed aggregates contain a large amount of *β*-folding structure. These type of diseases are commonly classified under the umbrella term of amyloidosis (31–33). Proteinaceous deposits with a starch-like appearance accumulate in various organs and tissues, leading to the formation of insoluble aggregates that are typically detectable through Congo red staining (34). Various factors, including genetic predisposition, environmental influences, and individual behavior, can contribute to the development of amyloidosis. In addition to traditional diagnostic methods, physicians may employ live tissue sectioning techniques to assess the presence of amyloid proteins within affected tissues (35). Although amyloid proteins possess similar pathogenic characteristics, their distribution within the body may differ, resulting in unique clinical presentations (36).

### 2.1 Islet amyloid polypeptide and T2DM

Diabetes mellitus is a chronic metabolic disease characterized by abnormally high blood glucose concentrations, and it is widely recognized that diabetes mellitus is genetically determined and triggered by acquired factors. Diabetes mellitus is often associated with serious complications, such as blindness, dyslipidemia, cardiovascular disease and renal failure. Currently, type 2 diabetes mellitus (T2DM) presents a significant global health concern, with the Centers for Disease Control and Prevention reporting approximately 40 million individuals in the United States affected by pre-diabetic conditions (37). A study conducted in the U.S. investigating the efficacy of lifestyle modifications or metformin in preventing the progression of impaired glucose tolerance (IGT) to diabetes revealed that 11% of individuals with IGT transition to diabetes annually, resulting in an estimated 2–4 million new cases of diabetes each year (38). A recent study predicts that the global prevalence of diabetes will increase from 2.8% in 2000 to 4.4% in 2030, meaning that nearly 366 million people will develop T2DM (39).

Human islet amyloid polypeptide is secreted by pancreatic islet β-cells and contains 37 amino acid residues, referred to as hIAPP37 (40). The aggregation and formation of amyloid fibril aggregates of human islet amyloid polypeptide have been shown to have deleterious effects on pancreatic islet cells, leading to damage and ultimately contributing to the development of T2DM (41, 42). The facile formation of toxic amyloid fibril aggregates is a prominent pathological characteristic of T2DM. After conducting numerous clinicopathologic experimental studies on patients with T2DM, it was determined that a significant proportion of individuals exhibited human pancreatic amyloid aggregates within the pancreatic islets, constituting approximately 90% of the total patient population. Conversely, human pancreatic islet amyloid aggregates were identified in only 15% of pancreatic islets in elderly individuals without diabetes mellitus symptoms (43).

Normally, insulin and islet amyloid polypeptides are co-secreted in response to blood glucose stimulation. In the atypical scenario of insulin resistance, the heightened demand for insulin prompts an elevation in insulin secretion, consequently resulting in an augmented co-secretion of islet amyloid polypeptide (44). It has been shown that the concentration of islet amyloid polypeptide in the blood during this process increases from the normal 1–20 pM to 50 pM (45), and the increased concentration puts the risk of generating amyloid deposits at an increased risk. The aggregates generated cause damage to the islet cells, which in turn exacerbates the insulin deficiency, and a vicious circle is set in motion.

### 2.2 *β*-amyloid peptide and AD

The AD is a neurodegenerative disease that occurs when the intelligence of the cerebral hemispheres of the brain, such as memory, language and reasoning, is in an impaired state (46). This degenerative brain disease, characterized by neurodementia, is considered a significant health concern impacting human longevity in contemporary society (47). Globally, the number of AD patients has now exceeded 30 million and is increasing by about 5 million per year, and it is expected that by 2040, the number of people suffering from AD will be more than 80 million, and the number in China will reach about 15 million, which is a three-fold increase compared with the beginning of this century (48). The disease is anticipated to impose significant economic strain and distress upon the families of afflicted individuals, thereby escalating into a pressing social issue of great concern.

The AD patients have two main types of abnormal lesions in the brain: (1) the presence of neurofibrillary tangles (NFTs) in the nerve cells of the brain, and (2) the appearance of age spots in the brain (49, 50). The primary components of the two lesions are abnormally phosphorylated Tau protein and *β* amyloid polypeptide, which is derived from the hydrolysis of an amyloid precursor protein called APP. APP contains a total of 770 amino acids, and its transmembrane region is capable of becoming a component of amyloid deposition through shearing (51). Another major pathology of NFTs is the formation of paired helical filaments (PHFs), which are mainly composed of hyperphosphorylated Tau protein (52). The number of NFTs in a patient’s brain has been reported to be a very important indicator of the severity of AD.

### 2.3 Nguyen virus protein and spongiform encephalopathy

Transmissible spongiform encephalopathies (TSEs) are fatal neurodegenerative diseases affecting both animals and humans, characterized by the presence of an infectious agent known as the self-expressed structurally abnormal Ranavirus protein (53). Examples of such diseases include Mad cow disease, Creutzfeldt-Jakob disease, and prurigo in sheep (54). The pathology of TSEs is characterized by the accumulation of amyloid plaque deposits, leading to degeneration, loss, vacuolar degeneration, death, and disappearance of neuronal cells in the cerebral cortex. This process ultimately results in the replacement of affected cells by vacuoles and stellate cells, causing a spongy state characterized by thinning of the cerebral cortex (gray matter) and relatively pronounced white matter, hence the term spongiform encephalopathy (55). Prion proteins (Prions) are a class of small molecule non-immunogenic hydrophobic proteins that can infect animals and replicate in host cells. The Prions molecule is incapable of inducing disease in its original form; rather, it must undergo a conformational change in order to adopt the infectious prion conformation and subsequently inflict damage upon neurons (56). Prions have the ability to induce conformational alterations in normal prion proteins, leading to their aggregation and subsequent interaction with additional normal prion proteins, ultimately resulting in a cascade effect that culminates in neuronal cell apoptosis (57). Normal Prion are present in both humans and animals and are labeled as PrP^c^ proteins, and Prions that are not normally present in the body but are infectious are labeled as PrP^sc^ (58). There is a specific hydrophobic sequence, the 106–126 fragment (denoted as PrP^106-126^), in the ryanovirus protein. This sequence has an important role in the conversion of PrP^C^ to PrP^SC^ and in the generation of associated neurotoxicity (59). PrP^106-126^ is frequently utilized as an *in vitro* model to investigating the aggregation process and pathomechanisms of Prions proteins due to its structural similarities with PrP^SC^, including a high prevalence of β-folded structures, propensity for aggregation to resist protease degradation, and significant cytotoxicity. To date, extensive research has been conducted on Prions and the PrP^106-126^ model; however, the precise mechanism underlying PrP^SC^-induced cytotoxicity and the pathogenic basis of aggregation remain elusive.

## 3 Amyloid fibrillation inhibition by natural polyphenols

**Figure 1:**
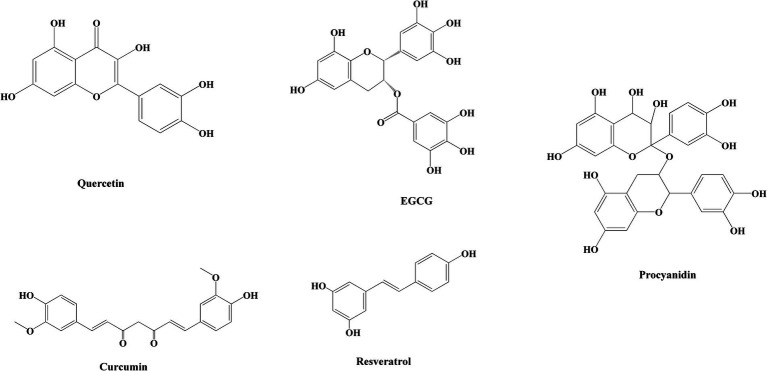
Chemical structures of polyphenols in common nanoparticles for inhibiting amyloid protein aggregation.

Toxic amyloid fibrils pose a significant threat to human health, yet a definitive treatment remains elusive. Up to now, researchers have screened dozens of inhibitors, which are broadly categorized into three groups: peptide inhibitors, antibodies, and small molecule inhibitors (60). Peptide inhibitors and antibodies offer the potential for targeted therapy; however, their limited utilization is primarily attributed to issues related to stability and cost. Small molecule inhibitors exhibit advantageous pharmacological properties and minimal cytotoxicity, with a wide range of natural polyphenol inhibitors standing out as particularly notable in this aspect (61, 62). The structural formulae of common polyphenols for inhibiting amyloid protein aggregation are depicted in Figure 1. Furthermore, certain natural polyphenols not only hinder amyloid fibril formation but also demonstrate neuroprotective properties, including the mitigation of neuroinflammation, resistance to oxidative stress and apoptosis, restoration of mitochondrial damage, and enhancement of fibril deposit clearance (63, 64).

Natural polyphenols have shown promising results in the treatment of a number of age-related diseases. Epigallocatechin gallate (EGCG) is a natural polyphenol compound found in green tea. It has been reported in the literature that the anti-amyloid effect of EGCG has been identified in SH-SY5Y neuronal cells and AD rat model in 2007 (65, 66). It has been shown that EGCG can convert the Aβ and *α*-synuclein touch nucleoprotein mature protofibrils and toxic oligomers into non-toxic small protein aggregates (67). Engel et al. (68) and Palhano et al. (69) have demonstrated that EGCG can inhibit hIAPP aggregation through hydrophobic effects. Resveratrol is also a natural polyphenol that is particularly abundant in grapes, and it is a natural non-flavonoid polyphenol that protects the cardiovascular system and prevent atherosclerosis (70). Resveratrol is also capable of directly interfering with amyloid aggregation of different peptides and reducing their toxicity (71). One study found that resveratrol effectively suppresses hIAPP aggregation via inhibiting the early formation of oligomeric intermediates (71). Resveratrol was also found to dose-dependently inhibit Aβ peptide amyloid fibrillation (72) and lysozyme amyloid fibrillation (73), and to break down already mature amyloid fibrils.

Curcumin, a polyphenol compound consisting of a *β*-diketone structure and two o-methylated phenols is derived from turmeric and has been utilized in traditional Chinese and Indian medicine for centuries (74). The significant curcumin content found in curry has been linked to a decreased risk of AD in the Indian population, as well as beneficial effects on cognitive function in elderly individuals (75). Traditional Chinese medicines such as turmeric (*Curcuma longa*) and *Poria cocos* contain abundant curcumin. Curcumin exhibits neuroprotective properties by inhibiting the aggregation of Aβ peptides, thereby altering the structure of Aβ fibrils and ameliorating the toxic effects induced by Aβ oligomers and the formation of non-toxic Aβ oligomers (76). Tannic acid possesses numerous hydroxyl and functional groups, enabling it to engage in interactions with diverse proteins, and these interactions can function as inhibitors of *β*-site amyloid precursor protein cleaving enzyme-1 (BACE1), Aβ, and Tau proteins (77).

## 4 Formation of polyphenol-based polymer nanoparticles

Polyphenols have garnered significant interest due to their distinctive physicochemical characteristics (78–81). Polyphenols can be categorized into different groups based on the number of phenolic rings they contain and the underlying structural elements that bind these rings, and can be classified into several subclasses such as phenolic acids, flavonoids, stilbenes, and lignans (82), where flavanones, flavonoids, flavonols, anthocyanins, and phenolic acids contain at least one benzene ring, aldehyde group, and several phenolic hydroxyls (83), which are essential structural components that facilitate the biological activity of polyphenols.

Numerous polyphenols exhibit limited water solubility and are vulnerable to environmental factors, leading to diminished bioavailability. The incorporation of composite nanoparticles has been shown to enhance the encapsulation efficiency of polyphenols and contribute to the improved stability of these bioactive compounds (84).

**Figure 2:**
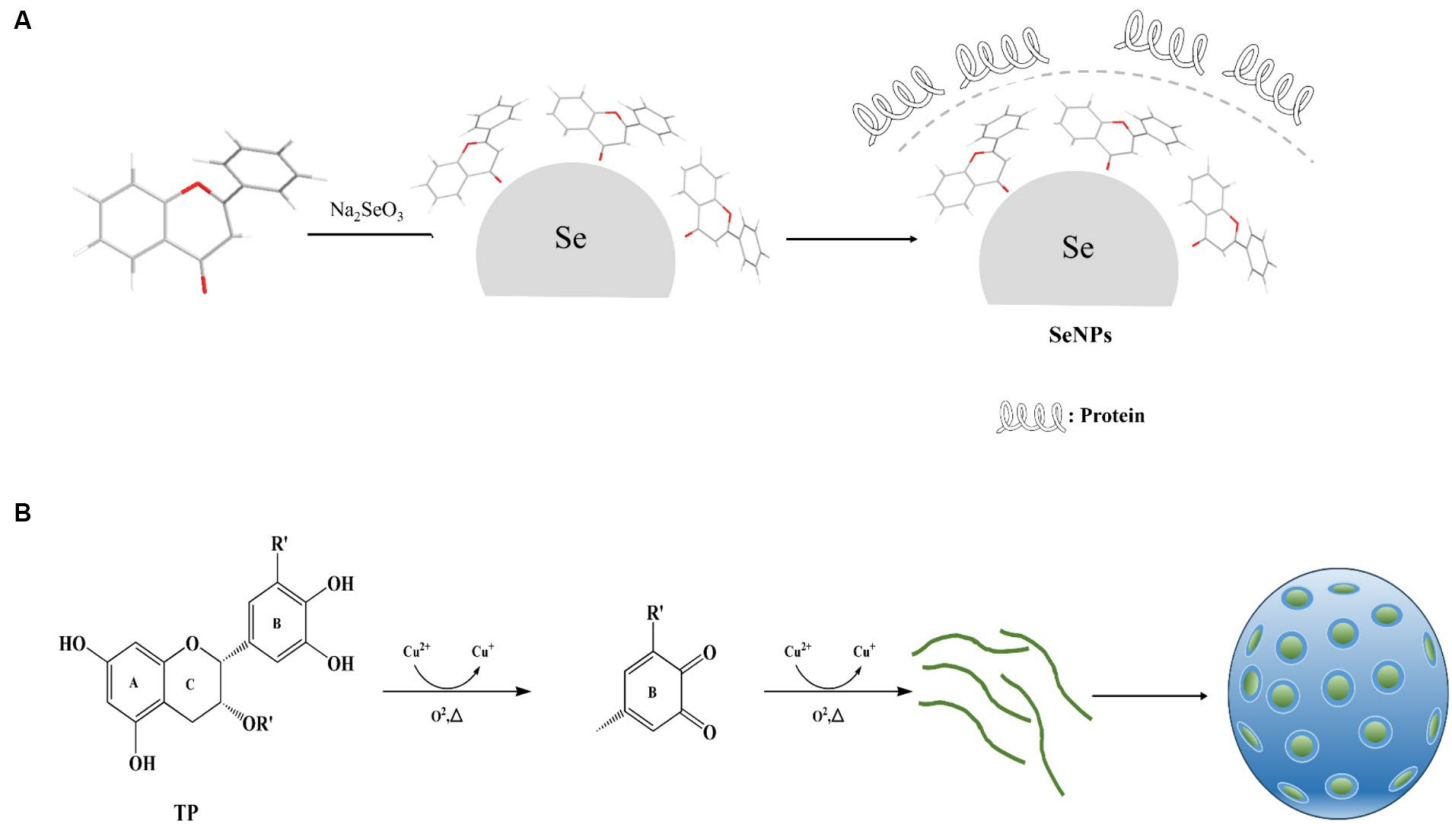
Mechanism of polyphenol nanoparticle formation. **(A)** Mechanism of Selenium Nanoparticle Formation. **(B)** Mechanism of Metal Oxide Nanoparticle Formation.

Polyphenols possess distinctive structural and chemical characteristics, notably the inclusion of functional groups such as catechol and gallophenol groups, enabling them to engage in diverse non-covalent and covalent interactions with a broad range of materials. These interactions make polyphenols applicable in various fields, encompassing inorganic materials like metal ions, metals, metal oxides, semiconductors, carbon, and silicon dioxide, as well as organic materials such as small molecules and synthetic polymers, and even bioactive biomolecules and active microorganisms (85–87) (Figure 2).

Such as, Sorasitthiyanukarn et al. (88) prepared chitosan/sodium alginate nanoparticles loaded with curcumin glutaric acid by O/W emulsification and ionization gelation, and optimized the conditions using response surface methodology, and the obtained products The study showed that curcumin glutaric acid-loaded chitosan/sodium alginate nanoparticles exhibited favorable stability, controlled release properties, and enhanced activity against multiple cancer cell lines. Zhou et al. (89) synthesized metal polyphenol nanoparticles using EGCG in aqueous solution with CuCl_2_ with low toxicity and high biocompatibility, improved lysosomal escape efficiency by doping with cell membrane-penetrating peptides, and achieved targeted delivery to mitochondria. In addition, Ma et al. (90) used Cu^2+^ chelated EGCG nanoparticles for the purpose of functionalizing collagen scaffolds into a spherical configuration. This structure exhibits slow release properties and possesses the capacity to scavenge free radicals, exhibit anti-inflammatory effects, inhibit bacterial growth, and promote angiogenesis.

## 5 Anti-amyloidogenic effects of polyphenol-based polymer nanoparticles

### 5.1 Green tea polyphenol-based nanoparticles

Green tea polyphenols consist of a diverse range of polyphenols present in green tea, with catechins constituting 70% ~ 80% of the overall polyphenol composition. The four main types of catechins found in green tea are epicatechin, epigallocatechin, epigallocatechin gallate, and EGCG. Among these, EGCG is the most abundant and bioactive compound in green tea, and has been extensively researched in inhibiting amyloid fibrillation and has been observed to reduce amyloid cytotoxicity induced by Huntington’s protein, alpha-synuclein, and Aβ (91–93). EGCG was found to directly bind to unfolded proteins and prevent the formation of β-sheet structures, which is an initial step in the cascade leading to amyloid formation. At the molecular level, autoxidized EGCG reacts with free primary amine groups of proteins to form Schiff bases and induce protofibril remodeling (94). The catechol structure of EGCG confers metal chelating, anti-inflammatory, antioxidant and neuroprotective activities that are crucial in the therapeutic management of AD (95). Debnath et al. (96) synthesized a 25 nm EGCG nanoparticle that was 10–100 times more efficient than native EGCG in inhibiting protein aggregation, breaking down mature protein aggregates, and reducing amyloidogenic cytotoxicity. This result suggests that EGCG-based polymer nanoparticles are more potent in preventing and treating protein aggregation-derived diseases. Zhang et al. (97) attached EGCG to selenium nanoparticles (EGCG@Se) and synthesized EGCG-stabilized selenium nanoparticles encapsulated with a Tet-1 peptide (Tet-1-EGCG@Se), EGCG@Se and Tet-1-EGCG@Se could label Aβ protofibrils with high affinity and the Tet-1 peptide could significantly enhance the cellular uptake of Tet-1-EGCG@Se in PC12 cells. In addition, studies have been conducted to prepare EGCG-derived carbonized polymer dots, and the prepared polymers interacted with Aβ through hydrogen bonding, electrostatic interactions, and hydrophobic interactions, leading to alterations in the Aβ aggregation pathway. It provides an important reference for the development of multifunctional EGCG-based polymer nanomaterials against neurodegenerative diseases and other protein conformation diseases (98).

### 5.2 Curcumin-based nanoparticles

Turmeric is widely produced in India as well as Pakistan, Bangladesh, China, Indonesia and Pakistan South America. The most active component of turmeric is curcumin, which makes up 2–5% of the spice (99). Numerous studies have demonstrated that curcumin can effectively inhibit amyloid fibrillogenesis. Such as, Pal et al. (100) explored the inhibition of amyloid fibrillogenesis by Al(III) and Zn(II) curcumin mixtures, and the results showed that metal-curcumin mixtures can inhibit the transition of oligomers to *β*-sheet protofibrils, and Al(III)-curcumin mixtures than Zn(II)-curcumin mixture was more effective in inhibiting *β*-protein aggregation. In addition, experimental evidence has shown that curcumin has the ability to inhibit the formation of Aβ amyloid fibrils by modifying the structure of protofibrils and altering the aggregation pathway of protofibrils (101).

Furthermore, native curcumin can inhibit the formation of amyloid fibrils by inhibiting the generation of primary nuclear structures of amyloid peptides through hydrogen bonding, hydrophobicity, and cationic tightness to A peptides (102). Brahmkhatri et al. (103) successfully synthesized curcumin-coated polymeric gold nanoparticles (PVP-C-AuNP) that demonstrated inhibition of Aβ_1-16_ aggregation by targeting the n-terminal region of Aβ amyloid peptide, and this study revealed that PVP-C-AuNP exhibited the ability to degrade mature amyloid fibrils. Mirzaie et al. (104) prepared methoxy polyethylene glycol polymer nano colloids with/without curcumin as phosphatidylethanolamine-stearoyl, and then explored their effects on the amyloid fibrillation process of bovine serum albumin, and the results showed that curcumin-loaded nano colloids had an inhibitory effect on the formation of amyloid fibrils. Research has also been conducted on the potential inhibition of amyloid fibrillation through structural modifications of curcumin. Solid lipid curcumin particles (SLCP) provide an alternative strategy for curcumin delivery, and intraperitoneal injection of SLCP in AD mice demonstrates superior therapeutic effectiveness compared to the free curcumin. Specifically, the lipid bilayer of SLCP facilitated crossing the blood–brain barrier. Within the brain, SLCP was found to bind to Aβ plaques in the prefrontal cortex and dentate gyrus, resulting in a decrease in the formation of Aβ_42_ oligomers and protofibrils, while also improving neuronal morphology (105). Giacomeli et al. (106) prepared curcumin-loaded lipid core nanocapsules (LNC) that demonstrated notable neuroprotective properties in mitigating Aβ_1-42_-induced behavioral and neurochemical alterations in an AD model.

### 5.3 Resveratrol-based nanoparticles

Resveratrol is a polyphenolic compound widely found in grapes, tiger nuts, peanuts, cassia and other plant foods or medicines, and is a fat-soluble plant antitoxin derived from plants (107, 108). Resveratrol exhibits a diverse array of physiological effects, including inhibition of cell membrane lipid peroxidation, protection against cardiovascular disease, anti-inflammatory properties, neuroprotection, and estrogenic activity (109, 110). Recently, nanoparticles have been used as effective carriers to enhance the oral bioavailability of resveratrol (111). Selenium nanoparticles functionalized on the surface of 100 nm resveratrol has been shown to be more effective than native resveratrol in inhibiting Aβ aggregation and reactive oxygen species (ROS) formation (112). Li et al. (113) prepared resveratrol-selenopeptide nanocomposites that interacted with Aβ, reduced Aβ aggregation, effectively inhibited Aβ deposition in the hippocampus, ameliorated cognitive deficits; and reduced Aβ-induced ROS and enhanced antioxidant enzyme activities in PC_12_ cells and *in vivo*; and also reduced Aβ-induced neuroinflammation in BV-2 cells and *in vivo* by regulating nuclear factor κB/mitogen-activated protein kinase/Akt signaling pathway to reduce Aβ-induced neuroinflammation in BV-2 cells and *in vivo*. Yang et al. (114) prepared resveratrol-loaded selenium nanoparticles/chitosan nanoparticles, which could alleviate cognitive deficits by restoring the balance of the intestinal flora, and thus suppress oxidative stress, neuroinflammation, and metabolic disorders in AD mice.

### 5.4 Anthocyanidin-based nanoparticles

Anthocyanidin are polyphenolic compounds found in fruits, grains, and flowers with antioxidant, anti-inflammatory, and anti-apoptotic properties (115, 116). Previous studies have shown that anthocyanins extracted from Korean black soybeans prevent neuroinflammation, neuronal apoptosis, and neuronal degeneration (117, 118). The findings indicate that anthocyanins may hold therapeutic potential for neurodegenerative disorder. Amin et al. (119) encapsulated anthocyanins in biodegradable nanoparticle formulations based on poly (lactide-*co*-glycolide) (PLGA) and stabilizer polyethylene glycol (PEG)-2000 delivery system. The findings demonstrate the therapeutic promise of anthocyanins in mitigating Alzheimer’s disease pathology and suggest that the efficacy of anthocyanins can be enhanced by utilizing nanomedicine delivery systems. Kim et al. (120) encapsulated anthocyanins in polyethylene glycol functionalized AuNPs, which were coupled with PEG AuNPs to enhance Aβ_1-4_ injecting the bioavailability of mice and controlling the release of anthocyanins. Kim et al. (120) prepared PEG-AuNPs loaded with anthocyanins that reduced Aβ_1-42_-induced markers of neuroinflammation and apoptosis through inhibition of the p-JNK/NF-κB/p-GSK3β pathway, and were more potent with PEG-AuNPs than with native anthocyanins alone. These results imply the possibility of PEG-coated AuNPs loaded with anthocyanins as therapeutic agents for neurodegenerative diseases, especially AD.

### 5.5 Quercetin-based nanoparticles

Quercetin is a flavonoid found in many vegetables and fruits and Traditional Chinese medicine such as white onion bulbs, lingonberries, cranberries, Kudzu root, and *Polygonum multiflorum* (121–123). Quercetin exhibits promise in attenuating the advancement of degenerative neurological disorders through the modulation of cellular pathways associated with Aβ-induced neurotoxicity and the alleviation of its adverse effects on neuronal cell lines and neurons (124–126). Nevertheless, the limited water insolubility, and extensive metabolism of quercetin pose challenges to its biological utility. A recent study has demonstrated that quercetin released from the nano-preparation maintains its biological activity by preparing quercetin-loaded modified core-shell mesoporous silica nano-preparations having a polyethylene glycol 3,000 surface-modified magnetite core that interferes with the aggregation of Aβ peptide and reduces the cytotoxicity of Aβ and the Aβ-induced generation of ROS (127). An additional method of encapsulating quercetin within lipid nanoparticles has been developed to produce a nano-system that is functionalized with transferrin, enabling the targeted delivery of quercetin to the brain. This approach has shown enhanced efficacy in inhibiting Aβ aggregation while maintaining minimal cytotoxic effects (128). The utilization of quercetin-based nanoparticle systems presents a novel approach to enhance current therapeutic interventions, offering compelling evidence and renewed optimism for the treatment of Alzheimer’s disease through quercetin administration.

## 6 Potential opportunities of polyphenol-based polymer nanoparticles

In recent years, nanotechnology-based pharmaceutical compounds delivery systems have received much attention for their potential to prolong drug residence and circulation in the bloodstream as well as to enhance the stability and solubility of pharmaceutical compounds. Advantages of the nanoparticle form include increased water solubility of amyloid-targeting molecules and enhanced binding affinity to amyloid structures (96). Optimal nanoparticle should possess non-toxic and biodegradable properties, with additional consideration given to factors such as material composition, preparation technique, nanoparticle dimensions, and surface modifications, all of which significantly impact the successful targeted delivery of pharmaceutical compounds, including penetration into the central nervous system. The potential function of nanoparticles is to stabilize labile pharmaceuticals, enhance their water solubility, prolong the presence of drugs or contrast agents in the circulatory system, and address the inherent limitations of pharmaceutical compounds (129). The application of nanotechnology in polyphenols delivery systems improves the bioavailability and kinetic properties of natural polyphenols in biological systems, and advances in nanotechnology help to target natural polyphenols to specific sites or molecular targets and deliver natural polyphenols safely to specific sites of action, especially for natural polyphenols targeting the central nervous system, such as for AD. The sustained release of polyphenol-based polymer nanoparticle enhances the controlled release properties of the loaded polyphenols, thereby minimizing the dosage regimen, minimizing the (toxic) side effects of the polyphenol, and maximizing the safety, which greatly improves the applicability and feasibility of the natural polyphenols.

Furthermore, the utilization of polyphenol-based polymer nanoparticles to target the central nervous system enhances the efficacy of natural polyphenols in traversing the blood–brain barrier, resulting in a synergistic therapeutic effect. Thus, the sustained effectiveness of natural polyphenol candidates in the treatment of AD can be enhanced by polyphenol-based polymer nanoparticles. Currently, chitosan, poly (alkyl cyanoacrylate) (PACA), PLGA and polyethylene glycol-modified PLGA (PEG-PLGA) are the most commonly used polymers for polyphenol-based polymer nanoparticles applications. PEG-PLGA are the most commonly used polymers in polyphenol-based polymer nanoparticles applications and play a great auxiliary role in brain-targeted delivery of natural polyphenols as well as in improving natural polyphenols utilization. Currently, it is also important to prepare safe and effective polyphenol-based polymer nanoparticles.

## 7 Conclusion and perspectives

Dr. Alois Alzheimer first described AD in 1906, but there are still no appropriate therapeutic drugs to treat it. Moreover, in the context of AD, existing treatments offer solely symptomatic relief and do not effectively halt the progression of the disease. No drug has been approved by the U.S. Food and Drug Administration for the treatment of AD since 2003. Therefore, it is important to improve the efficacy of currently available pharmaceutical compounds through the use of delivery technologies including nanomedicines and to develop new polymer nanoparticles that block all possible mechanisms of disease pathogenesis in order to treat patients with AD.

**Figure 3:**
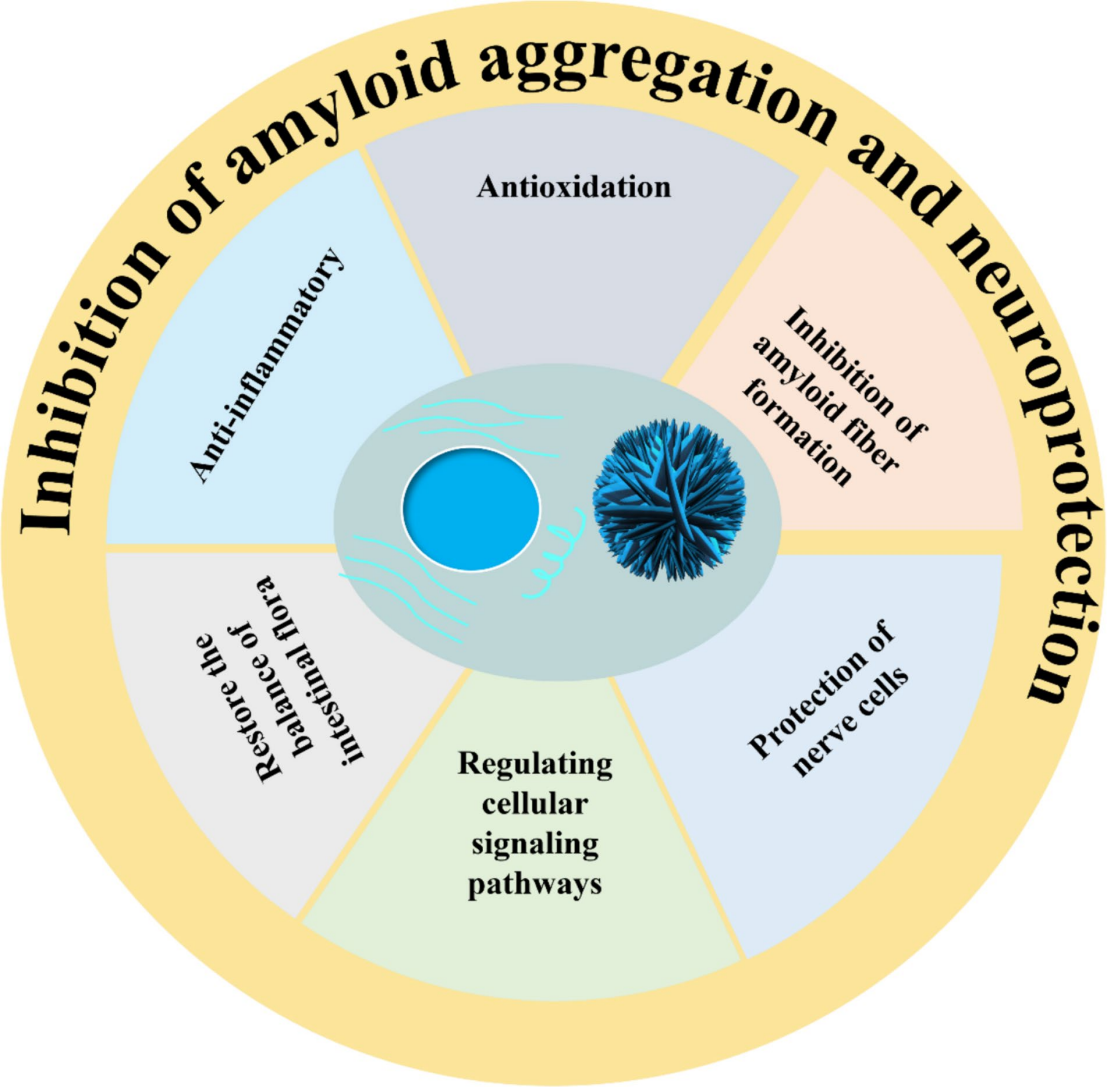
Pathways of polyphenols in inhibiting amyloid deposition and neuroprotective effect.

Natural polyphenols are widely found in plants in nature. Due to their diverse biological activities, including antioxidant, anti-inflammatory, and intestinal flora regulation properties, they are utilized in the food industry as primary nutritional components in functional foods (Figure 3). Numerous studies have shown that natural polyphenols effectively mitigate the deleterious impact of amyloid peptides on neuronal cells and neurons; however, these compounds are constrained by various limitations including the necessity for higher doses to achieve therapeutic efficacy, suboptimal absorption rates, restricted bioavailability, peripheral side effects, and challenges in traversing the blood–brain barrier. Natural polyphenols have the chemical property of providing both covalent and non-covalent bonds, and thus they have great potential for engineering materials applications to construct inorganic compound-phenolic fractions while generating functional polyphenol-based inorganic hybrid nanoparticles. In addition, by controlling and stabilizing the physicochemical interactions between molecules, polyphenols can not only self-assemble into particles in confined spaces, but also form coatings on the surfaces of preformed particles or serve as templates for the secondary growth of other functional materials. Thus, having special physicochemical properties makes polyphenols of practical use in various fields of polymer nanoparticles application.

**Figure 4:**
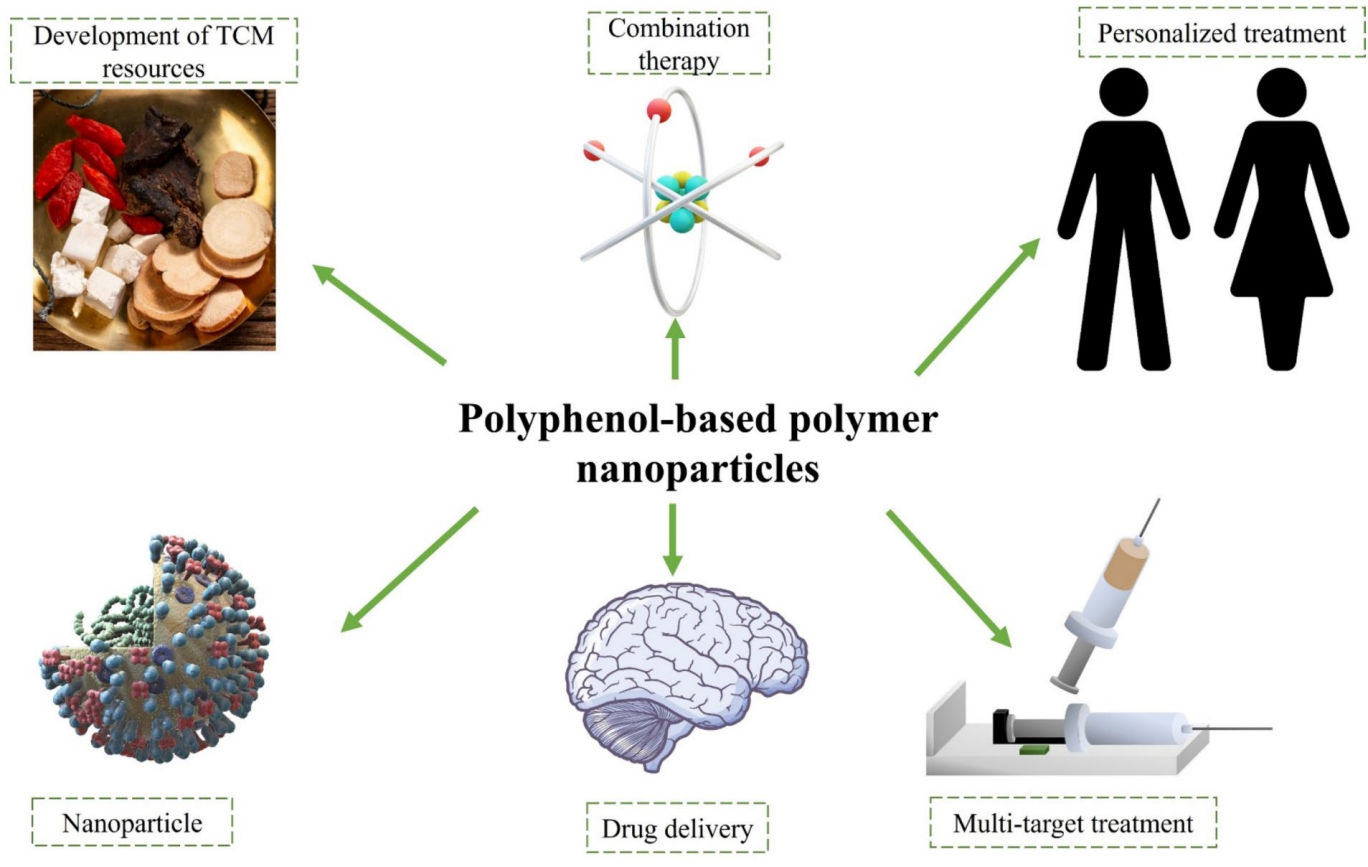
Perspectives on polyphenolic polymer nanoparticles in inhibiting amyloid aggregation in the field of traditional Chinese medicine.

The research on polyphenol-based polymer nanoparticles in inhibiting amyloid protein aggregation in the field of traditional Chinese medicine (TCM) also holds great potential (Figure 4). The major manifestations include: (1) development of TCM resources: TCM possesses abundant plant resources, many of which are rich in polyphenol. Through research on polyphenol in TCM, it is possible to develop polyphenol-based polymer nanoparticles that possess the capability to inhibit amyloid protein aggregation. (2) Application of polyphenol-based nanoparticles: TCM ingredients have been proven to possess the efficacy of inhibiting amyloid protein aggregation. The enhancement of therapeutic effects of TCM can be achieved through the preparation of polyphenol-based nanoparticles, which serve to improve the bioavailability and stability of the medications. (3) Compatibility application of TCM polyphenol-based nanoparticles: TCM emphasizes the compatibility of medications, aiming to enhance therapeutic effects through the combination of multiple pharmaceutical compounds. The incorporation of polyphenol-based polymer nanoparticles in conjunction with other TCM components has been shown to augment the suppression of amyloid protein aggregation. (4) TCM’s multi-target treatment strategy: TCM focuses on the holistic concept and adopts a multi-target treatment strategy. Polyphenol-based polymer nanoparticles can interact with multiple targets, inhibiting abnormal aggregation of amyloid proteins and intervening in diseases related to amyloid proteins at multiple levels. (5) Personalized treatment: TCM emphasizes personalized treatment, providing targeted therapies based on the patient’s constitution and condition. Polyphenol-based polymer nanoparticles can be customized according to the specific situation of the patient, offering personalized treatment plans.

Although polyphenol-based polymer nanoparticles have shown potential in inhibiting amyloid protein aggregation in the field of TCM, further research and clinical experiments are still needed to verify their safety and effectiveness. Simultaneously, further research is needed to investigate the integration of TCM with modern scientific principles in order to advance the utilization of polyphenolic compounds for inhibiting amyloid protein aggregation in therapeutic applications.

## Author contributions

SF: Conceptualization, Investigation, Methodology, Validation, Writing – original draft, Writing – review & editing. KZ: Formal analysis, Software, Validation, Writing – review & editing. DL: Formal analysis, Methodology, Writing – review & editing. YY: Formal analysis, Writing – review & editing. HX: Methodology, Writing – review & editing. WX: Formal analysis, Writing – review & editing. KD: Funding acquisition, Writing – review & editing. ZR: Resources, Writing – review & editing. DW: Methodology, Supervision, Writing – review & editing. WY: Conceptualization, Funding acquisition, Resources, Supervision, Writing – review & editing.

## References

[1] 2021 Alzheimer’s disease facts and figures. Alzheimers Dement. (2021) 17:327–406. doi: 10.1002/alz.12328 33756057

[2] Golde TE Levey AI. Immunotherapies for Alzheimer's disease. Science. (2023) 382:1242–4. doi: 10.1126/science.adj9255 38096276

[3] Mayeux R. Alzheimer's disease biomarkers – timing is everything. N Engl J Med. (2024) 390:761–3. doi: 10.1056/NEJMe2400102, PMID: 38381680

[4] Zhao Y Deng H Li K Wang L Wu Y Dong X. Trans-cinnamaldehyde improves neuroinflammation-mediated NMDA receptor dysfunction and memory deficits through blocking NF-kappaB pathway in presenilin1/2 conditional double knockout mice. Brain Behav Immun. (2019) 82:45–62. doi: 10.1016/j.bbi.2019.07.032, PMID: 31376499

[5] Bateman RJ Smith J Donohue MC Delmar P Abbas R Salloway S. Two phase 3 trials of Gantenerumab in early Alzheimer's disease. N Engl J Med. (2023) 389:1862–76. doi: 10.1056/NEJMoa2304430 37966285 PMC10794000

[6] Jia J Ning Y Chen M Wang S Yang H Li F. Biomarker changes during 20 years preceding Alzheimer's disease. N Engl J Med. (2024) 390:712–22. doi: 10.1056/NEJMoa2310168, PMID: 38381674

[7] Lee SJ Nam E Lee HJ Savelieff MG Lim MH. Towards an understanding of amyloid-beta oligomers: characterization, toxicity mechanisms, and inhibitors. Chem Soc Rev. (2017) 46:310–23. doi: 10.1039/C6CS00731G, PMID: 27878186

[8] Liu H Qian C Yang T Wang Y Luo J Zhang C. Small molecule-mediated co-assembly of amyloid-beta oligomers reduces neurotoxicity through promoting non-fibrillar aggregation. Chem Sci. (2020) 11:7158–69. doi: 10.1039/D0SC00392A, PMID: 34123000 PMC8159368

[9] Huang Y Guo X Wu Y Chen X Feng L Xie N. Nanotechnology's frontier in combatting infectious and inflammatory diseases: prevention and treatment. Signal Transduct Target Ther. (2024) 9:34. doi: 10.1038/s41392-024-01745-z, PMID: 38378653 PMC10879169

[10] Zou J Zhang Y Pan Y Mao Z Chen X. Advancing nanotechnology for neoantigen-based cancer theranostics. Chem Soc Rev. (2024) 53:3224–52. doi: 10.1039/D3CS00162H 38379286

[11] Grams RJ Santos WL Scorei IR Abad-Garcia A Rosenblum CA Bita A. The rise of boron-containing compounds: advancements in synthesis, medicinal chemistry, and emerging pharmacology. Chem Rev. (2024) 124:2441–511. doi: 10.1021/acs.chemrev.3c00663, PMID: 38382032

[12] Qi Y Xu C Zhang Z Zhang Q Xu Z Zhao X. Wet environment-induced adhesion and softening of coenzyme-based polymer elastic patch for treating periodontitis. Bioact Mater. (2024) 35:259–73. doi: 10.1016/j.bioactmat.2024.02.002, PMID: 38356924 PMC10864166

[13] Wang Z Yang F Liu X Han X Li X Huyan C. Hydrogen bonds-pinned entanglement blunting the interfacial crack of hydrogel-elastomer hybrids. Adv Mater. (2024) 36:3177. doi: 10.1002/adma.202313177 38272488

[14] Monahan M Homer M Zhang S Zheng R Chen CL De Yoreo J. Impact of nanoparticle size and surface chemistry on Peptoid self-assembly. ACS Nano. (2022) 16:8095–106. doi: 10.1021/acsnano.2c01203, PMID: 35486471

[15] Neal TJ Penfold NJW Armes SP. Reverse sequence polymerization-induced self-assembly in aqueous media. Angew Chem Int Ed Engl. (2022) 61:e202207376. doi: 10.1002/anie.202207376, PMID: 35678548 PMC9541501

[16] Lenders V Koutsoumpou X Phan P Soenen SJ Allegaert K De Vleeschouwer S. Modulation of engineered nanomaterial interactions with organ barriers for enhanced drug transport. Chem Soc Rev. (2023) 52:4672–724. doi: 10.1039/D1CS00574J, PMID: 37338993

[17] Sela M Poley M Mora-Raimundo P Kagan S Avital A Kaduri M. Brain-targeted liposomes loaded with monoclonal antibodies reduce alpha-Synuclein aggregation and improve behavioral symptoms in Parkinson's disease. Adv Mater. (2023) 35:e2304654. doi: 10.1002/adma.202304654 37753928 PMC7615408

[18] Shah MA Faheem HI Hamid A Yousaf R Haris M Saleem U. The entrancing role of dietary polyphenols against the most frequent aging-associated diseases. Med Res Rev. (2024) 44:235–74. doi: 10.1002/med.21985, PMID: 37486109

[19] Yang P Huang Q Zhang J Li Y Gao H Gu Z. Natural polyphenolic nanodots for Alzheimer's disease treatment. Adv Mater. (2024) 36:e2308393. doi: 10.1002/adma.202308393 38010256

[20] Laurindo LF De Carvalho GM De Oliveira Zanuso B Figueira ME Direito R De Alvares Goulart R. Curcumin-based Nanomedicines in the treatment of inflammatory and immunomodulated diseases: an Evidence-Based Comprehensive Review. Pharmaceutics. (2023) 15:229. doi: 10.3390/pharmaceutics15010229 36678859 PMC9861982

[21] Szymczak J Cielecka-Piontek J. Fisetin-in search of better bioavailability-from macro to Nano modifications: a review. Int J Mol Sci. (2023) 24:14158. doi: 10.3390/ijms241814158, PMID: 37762460 PMC10532335

[22] Sabatini PV Lynn FC. All-encomPASsing regulation of beta-cells: PAS domain proteins in beta-cell dysfunction and diabetes. Trends Endocrinol Metab. (2015) 26:49–57. doi: 10.1016/j.tem.2014.11.002, PMID: 25500169

[23] Song Y Li P Liu L Bortolini C Dong M. Nanostructural differentiation and toxicity of amyloid-beta25-35 aggregates ensue from distinct secondary conformation. Sci Rep. (2018) 8:765. doi: 10.1038/s41598-017-19106-y, PMID: 29335442 PMC5768673

[24] Song Y Li P Zhang Z Wang Y Zhang Z Liu L. Photodegradation of porphyrin-bound hIAPP(1–37) fibrils. New J Chem. (2020) 44:9438–43. doi: 10.1039/C9NJ06082K

[25] Canu N Amadoro G Triaca V Latina V Sposato V Corsetti V. The intersection of NGF/TrkA signaling and amyloid precursor protein processing in Alzheimer's disease neuropathology. Int J Mol Sci. (2017) 18:1319. doi: 10.3390/ijms18061319, PMID: 28632177 PMC5486140

[26] Spires-Jones TL Hyman BT. The intersection of amyloid beta and tau at synapses in Alzheimer's disease. Neuron. (2014) 82:756–71. doi: 10.1016/j.neuron.2014.05.004, PMID: 24853936 PMC4135182

[27] Dehay B Bourdenx M Gorry P Przedborski S Vila M Hunot S. Targeting alpha-synuclein for treatment of Parkinson's disease: mechanistic and therapeutic considerations. Lancet Neurol. (2015) 14:855–66. doi: 10.1016/S1474-4422(15)00006-X, PMID: 26050140 PMC5217462

[28] Griffey CJ Yamamoto A. Living in alpha-syn: tackling aggregates in Parkinson's disease. Neuron. (2022) 110:351–2. doi: 10.1016/j.neuron.2022.01.016, PMID: 35114103

[29] Lazarov O Mattson MP Peterson DA Pimplikar SW Van Praag H. When neurogenesis encounters aging and disease. Trends Neurosci. (2010) 33:569–79. doi: 10.1016/j.tins.2010.09.003, PMID: 20961627 PMC2981641

[30] Amini AA Solovyova AS Sadeghian H Blackbourn DJ Rezaee SA. Structural properties of a viral orthologue of cellular CD200 protein: KSHV vOX2. Virology. (2015) 474:94–104. doi: 10.1016/j.virol.2014.10.020, PMID: 25463607

[31] Ke PC Zhou R Serpell LC Riek R Knowles TPJ Lashuel HA. Half a century of amyloids: past, present and future. Chem Soc Rev. (2020) 49:5473–509. doi: 10.1039/C9CS00199A, PMID: 32632432 PMC7445747

[32] Knowles TP Vendruscolo M Dobson CM. The amyloid state and its association with protein misfolding diseases. Nat Rev Mol Cell Biol. (2014) 15:384–96. doi: 10.1038/nrm3810 24854788

[33] Schweighauser M Arseni D Bacioglu M Huang M Lovestam S Shi Y. Age-dependent formation of TMEM106B amyloid filaments in human brains. Nature. (2022) 605:310–4. doi: 10.1038/s41586-022-04650-z, PMID: 35344985 PMC9095482

[34] Wilcock DM Gordon MN Morgan D. Quantification of cerebral amyloid angiopathy and parenchymal amyloid plaques with Congo red histochemical stain. Nat Protoc. (2006) 1:1591–5. doi: 10.1038/nprot.2006.277, PMID: 17406451

[35] Hadley KC Borrelli MJ Lepock JR Mclaurin J Croul SE Guha A. Multiphoton ANS fluorescence microscopy as an in vivo sensor for protein misfolding stress. Cell Stress Chaperones. (2011) 16:549–61. doi: 10.1007/s12192-011-0266-6, PMID: 21484286 PMC3156256

[36] Mccollum LE Das SR Xie L De Flores R Wang J Xie SX. Oh brother, where art tau? Amyloid, neurodegeneration, and cognitive decline without elevated tau. Neuroimage Clin. (2021) 31:102717. doi: 10.1016/j.nicl.2021.102717, PMID: 34119903 PMC8207301

[37] American Diabetes A. Economic costs of diabetes in the U.S. in 2012. Diabetes Care. (2013) 36:1033–46. doi: 10.2337/dc12-2625, PMID: 23468086 PMC3609540

[38] Knowler WC Barrett-Connor E Fowler SE Hamman RF Lachin JM Walker EA. Reduction in the incidence of type 2 diabetes with lifestyle intervention or metformin. N Engl J Med. (2002) 346:393–403. doi: 10.1056/NEJMoa012512 11832527 PMC1370926

[39] GBD 2021 Diseases and Injuries Collaborators. Global incidence, prevalence, years lived with disability (YLDs), disability-adjusted life-years (DALYs), and healthy life expectancy (HALE) for 371 diseases and injuries in 204 countries and territories and 811 subnational locations, 1990-2021: a systematic analysis for the Global Burden of Disease Study 2021. Lancet. (2024) 403,10440:2133–61. doi: 10.1016/S0140-6736(24)00757-8 38642570 PMC11122111

[40] Bortoletto AS Graham WV Trout G Bonito-Oliva A Kazmi MA Gong J. Human islet amyloid polypeptide (hIAPP) Protofibril-specific antibodies for detection and treatment of type 2 Diabetes. Adv Sci. (2022) 9:e2202342. doi: 10.1002/advs.202202342 PMC9731688 36257905

[41] Szunerits S Abderrahmani A Boukherroub R. Nanoparticles and nanocolloidal carbon: will they be the next antidiabetic class that targets fibrillation and aggregation of human islet amyloid polypeptide in type 2 Diabetes? Acc Chem Res. (2022) 55:2869–81. doi: 10.1021/acs.accounts.2c00415, PMID: 36174237

[42] Xin Y Wang S Liu H Ke H Tian S Cao Y. Hierarchical vitalization of oligotyrosine in mitigating islet amyloid polypeptide amyloidogenesis through multivalent macromolecules with conformation-restrained Nanobody ligands. ACS Nano. (2021) 15:13319–28. doi: 10.1021/acsnano.1c03083, PMID: 34293858

[43] Westermark P. Quantitative studies on amyloid in the islets of Langerhans. Ups J Med Sci. (1972) 77:91–4. doi: 10.1517/03009734000000014 4116019

[44] Akter R Cao P Noor H Ridgway Z Tu LH Wang H. Islet amyloid polypeptide: structure, function, and pathophysiology. J Diabetes Res. (2016) 2016:1–18. doi: 10.1155/2016/2798269 PMC4662979 26649319

[45] Masad A Tabner BJ Mayes J Allsop D. The amylin peptide implicated in type 2 diabetes stimulates copper-mediated carbonyl group and ascorbate radical formation. Free Radic Biol Med. (2011) 51:869–75. doi: 10.1016/j.freeradbiomed.2011.05.033, PMID: 21683137

[46] Bakhta K Cecillon E Lacombe E Lamy M Leboucher A Philippe J. Alzheimer's disease and neurodegenerative diseases in France. Lancet. (2019) 394:466–7. doi: 10.1016/S0140-6736(19)31633-2 31402022

[47] Faissner S Plemel JR Gold R Yong VW. Progressive multiple sclerosis: from pathophysiology to therapeutic strategies. Nat Rev Drug Discov. (2019) 18:905–22. doi: 10.1038/s41573-019-0035-2, PMID: 31399729

[48] 2023 Alzheimer’s disease facts and figures. Alzheimers Dementia. (2023) 19:1598–695. 10.1002/alz.13016 36918389

[49] Blennow K Mattsson N Scholl M Hansson O Zetterberg H. Amyloid biomarkers in Alzheimer's disease. Trends Pharmacol Sci. (2015) 36:297–309. doi: 10.1016/j.tips.2015.03.002 25840462

[50] Filippi M Cecchetti G Spinelli EG Vezzulli P Falini A Agosta F. Amyloid-related imaging abnormalities and beta-amyloid-targeting antibodies: a systematic review. JAMA Neurol. (2022) 79:291–304. doi: 10.1001/jamaneurol.2021.5205, PMID: 35099507

[51] Mustafa MK Nabok A Parkinson D Tothill IE Salam F Tsargorodskaya A. Detection of beta-amyloid peptide (1-16) and amyloid precursor protein (APP770) using spectroscopic ellipsometry and QCM techniques: a step forward towards Alzheimers disease diagnostics. Biosens Bioelectron. (2010) 26:1332–6. doi: 10.1016/j.bios.2010.07.042, PMID: 20692146

[52] Karran E De Strooper B. The amyloid hypothesis in Alzheimer disease: new insights from new therapeutics. Nat Rev Drug Discov. (2022) 21:306–18. doi: 10.1038/s41573-022-00391-w, PMID: 35177833

[53] Collins SJ Lawson VA Masters CL. Transmissible spongiform encephalopathies. Lancet. (2004) 363:51–61. doi: 10.1016/S0140-6736(03)15171-9 14723996

[54] Dobson CM. The structural basis of protein folding and its links with human disease. Philos Trans R Soc Lond Ser B Biol *Sci*. (2001) 356:133–45. doi: 10.1098/rstb.2000.0758, PMID: 11260793 PMC1088418

[55] Sigurdson CJ Bartz JC Glatzel M. Cellular and molecular mechanisms of prion disease. Annu Rev Pathol. (2019) 14:497–516. doi: 10.1146/annurev-pathmechdis-012418-013109, PMID: 30355150 PMC9071098

[56] Frontzek K Bardelli M Senatore A Henzi A Reimann RR Bedir S. A conformational switch controlling the toxicity of the prion protein. Nat Struct Mol Biol. (2022) 29:831–40. doi: 10.1038/s41594-022-00814-7, PMID: 35948768 PMC9371974

[57] Resenberger UK Harmeier A Woerner AC Goodman JL Muller V Krishnan R. The cellular prion protein mediates neurotoxic signalling of beta-sheet-rich conformers independent of prion replication. EMBO J. (2011) 30:2057–70. doi: 10.1038/emboj.2011.86, PMID: 21441896 PMC3098494

[58] Aguzzi A De Cecco E. Shifts and drifts in prion science. Science. (2020) 370:32–4. doi: 10.1126/science.abb8577, PMID: 33004500

[59] Li B Xu C Lv Y Liu G Sun X Sun Z. Vanadium-substituted polyoxometalates regulate prion protein fragment 106-126 misfolding by an oxidation strategy. ACS Appl Mater Interfaces. (2023) 15:34497–504. doi: 10.1021/acsami.3c04969, PMID: 37439628

[60] Sievers SA Karanicolas J Chang HW Zhao A Jiang L Zirafi O. Structure-based design of non-natural amino-acid inhibitors of amyloid fibril formation. Nature. (2011) 475:96–100. doi: 10.1038/nature10154, PMID: 21677644 PMC4073670

[61] Han J Du Z Lim MH. Mechanistic insight into the Design of Chemical Tools to control multiple pathogenic features in Alzheimer's disease. Acc Chem Res. (2021) 54:3930–40. doi: 10.1021/acs.accounts.1c00457, PMID: 34606227

[62] Young LM Saunders JC Mahood RA Revill CH Foster RJ Tu LH. Screening and classifying small-molecule inhibitors of amyloid formation using ion mobility spectrometry-mass spectrometry. Nat Chem. (2015) 7:73–81. doi: 10.1038/nchem.2129, PMID: 25515893 PMC4280571

[63] Gao M Zhao Z Lv P Li Y Gao J Zhang M. Quantitative combination of natural anti-oxidants prevents metabolic syndrome by reducing oxidative stress. Redox Biol. (2015) 6:206–17. doi: 10.1016/j.redox.2015.06.013, PMID: 26262997 PMC4536297

[64] Thummayot S Tocharus C Jumnongprakhon P Suksamrarn A Tocharus J. Cyanidin attenuates Abeta(25-35)-induced neuroinflammation by suppressing NF-kappaB activity downstream of TLR4/NOX4 in human neuroblastoma cells. Acta Pharmacol Sin. (2018) 39:1439–52. doi: 10.1038/aps.2017.203, PMID: 29671417 PMC6289386

[65] Jang S You K-W Kim SH Park S-J Park J-S Jeong H-S. Protein kinase C-mediated neuroprotective action of (−)-epigallocatechin-3-gallate against Aβ1-42-induced apoptotic cell death in SH-SY5Y Neuroblastoma cells. Korean J Physiol Pharmacol. (2007) 11:163–9.

[66] Rasoolijazi H Joghataie MT Roghani M Nobakht M. The beneficial effect of (−)-epigallocatechin-3-gallate in an experimental model of Alzheimer's disease in rat: a behavioral analysis. Iran Biomed J. (2007) 11:237–43. PMID: 18392085

[67] Yoshida W Kobayashi N Sasaki Y Ikebukuro K Sode K. Partial peptide of alpha-synuclein modified with small-molecule inhibitors specifically inhibits amyloid fibrillation of alpha-synuclein. Int J Mol Sci. (2013) 14:2590–600. doi: 10.3390/ijms14022590, PMID: 23358249 PMC3588004

[68] Engel MF Vandenakker CC Schleeger M Velikov KP Koenderink GH Bonn M. The polyphenol EGCG inhibits amyloid formation less efficiently at phospholipid interfaces than in bulk solution. J Am Chem Soc. (2012) 134:14781–8. doi: 10.1021/ja3031664, PMID: 22889183

[69] Palhano FL Lee J Grimster NP Kelly JW. Toward the molecular mechanism(s) by which EGCG treatment remodels mature amyloid fibrils. J Am Chem Soc. (2013) 135:7503–10. doi: 10.1021/ja3115696, PMID: 23611538 PMC3674815

[70] Cheng CK Luo JY Lau CW Chen ZY Tian XY Huang Y. Pharmacological basis and new insights of resveratrol action in the cardiovascular system. Br J Pharmacol. (2020) 177:1258–77. doi: 10.1111/bph.14801, PMID: 31347157 PMC7056472

[71] Stefani M Rigacci S. Protein folding and aggregation into amyloid: the interference by natural phenolic compounds. Int J Mol Sci. (2013) 14:12411–57. doi: 10.3390/ijms140612411, PMID: 23765219 PMC3709793

[72] Chan S Kantham S Rao VM Palanivelu MK Pham HL Shaw PN. Metal chelation, radical scavenging and inhibition of Abeta(4)(2) fibrillation by food constituents in relation to Alzheimer's disease. Food Chem. (2016) 199:185–94. doi: 10.1016/j.foodchem.2015.11.118, PMID: 26775960

[73] Shariatizi S Meratan AA Ghasemi A Nemat-Gorgani M. Inhibition of amyloid fibrillation and cytotoxicity of lysozyme fibrillation products by polyphenols. Int J Biol Macromol. (2015) 80:95–106. doi: 10.1016/j.ijbiomac.2015.06.030, PMID: 26102331

[74] Hatcher H Planalp R Cho J Torti FM Torti SV. Curcumin: from ancient medicine to current clinical trials. Cell Mol Life Sci. (2008) 65:1631–52. doi: 10.1007/s00018-008-7452-4, PMID: 18324353 PMC4686230

[75] Ng TP Nyunt MSZ Gao Q Gwee X Chua DQL Yap KB. Curcumin-rich curry consumption and neurocognitive function from 4.5-year follow-up of community-dwelling older adults (Singapore longitudinal ageing study). Nutrients. (2022) 14:1189. doi: 10.3390/nu14061189, PMID: 35334842 PMC8952785

[76] Marques MS Marinho MAG Vian CO And Horn AP. The action of curcumin against damage resulting from cerebral stroke: a systematic review. Pharmacol Res. (2022) 183:106369. doi: 10.1016/j.phrs.2022.106369, PMID: 35914679

[77] Sylla T Pouysegu L Da Costa G Deffieux D Monti JP Quideau S. Gallotannins and tannic acid: first chemical syntheses and in vitro inhibitory activity on Alzheimer's amyloid beta-peptide aggregation. Angew Chem Int Ed Engl. (2015) 54:8217–21. doi: 10.1002/anie.201411606, PMID: 26013280

[78] Chiorcea-Paquim AM Enache TA De Souza Gil E Oliveira-Brett AM. Natural phenolic antioxidants electrochemistry: towards a new food science methodology. Compr Rev Food Sci Food Saf. (2020) 19:1680–726. doi: 10.1111/1541-4337.12566, PMID: 33337087

[79] Quideau S Deffieux D Douat-Casassus C Pouysegu L. Plant polyphenols: chemical properties, biological activities, and synthesis. Angew Chem Int Ed Engl. (2011) 50:586–621. doi: 10.1002/anie.201000044 21226137

[80] Ren Z Sun S Sun R Cui G Hong L Rao B. A metal-polyphenol-coordinated nanomedicine for synergistic Cascade Cancer chemotherapy and chemodynamic therapy. Adv Mater. (2020) 32:e1906024. doi: 10.1002/adma.201906024 31834662

[81] Yang T Li C Xue W Huang L Wang Z. Natural immunomodulating substances used for alleviating food allergy. Crit Rev Food Sci Nutr. (2023) 63:2407–25. doi: 10.1080/10408398.2021.1975257, PMID: 34494479

[82] Yan X Zeng Z Mcclements DJ Gong X Yu P Xia J. A review of the structure, function, and application of plant-based protein-phenolic conjugates and complexes. Compr Rev Food Sci Food Saf. (2023) 22:1312–36. doi: 10.1111/1541-4337.13112, PMID: 36789802

[83] Chen Z Farag MA Zhong Z Zhang C Yang Y Wang S. Multifaceted role of phyto-derived polyphenols in nanodrug delivery systems. Adv Drug Deliv Rev. (2021) 176:113870. doi: 10.1016/j.addr.2021.113870, PMID: 34280511

[84] Li H Song J Liu C Wang X Liu Y Han M. Corn starch/β-cyclodextrin composite nanoparticles for encapsulation of tea polyphenol and development of oral targeted delivery systems with pH-responsive properties. Food Hydrocoll. (2024) 151:109823. doi: 10.1016/j.foodhyd.2024.109823

[85] Han Y Lin Z Zhou J Yun G Guo R Richardson JJ. Polyphenol-mediated assembly of proteins for engineering functional materials. Angew Chem Int Ed Engl. (2020) 59:15618–25. doi: 10.1002/anie.202002089 32115863

[86] Zhou J Lin Z Ju Y Rahim MA Richardson JJ Caruso F. Polyphenol-mediated assembly for particle engineering. Acc Chem Res. (2020) 53:1269–78. doi: 10.1021/acs.accounts.0c00150, PMID: 32567830

[87] Zhou J Lin Z Penna M Pan S Ju Y Li S. Particle engineering enabled by polyphenol-mediated supramolecular networks. Nat Commun. (2020) 11:4804. doi: 10.1038/s41467-020-18589-0 32968077 PMC7511334

[88] Sorasitthiyanukarn FN Muangnoi C Bhuket RN Rojsitthisak P Rojsitthisak P. Chitosan/alginate nanoparticles as a promising approach for oral delivery of curcumin diglutaric acid for cancer treatment. Mater Sci Eng C Mater Biol Appl. (2018) 93:178–90. doi: 10.1016/j.msec.2018.07.069, PMID: 30274050

[89] Zhou H Qian Q Chen Q Chen T Wu C Chen L. Enhanced mitochondrial targeting and inhibition of pyroptosis with multifunctional metallopolyphenol nanoparticles in intervertebral disc degeneration. Small. (2023) 20:e2308167. doi: 10.1002/smll.202308167 37953455

[90] Ma L Tan Y Tong Q Cao X Liu D Ma X. Collagen scaffolds functionalized by cu(2+) -chelated EGCG nanoparticles with anti-inflammatory, anti-oxidation, vascularization, and anti-bacterial activities for accelerating wound healing. Adv Healthc Mater. (2024) 13:e2303297. doi: 10.1002/adhm.202303297 38315874

[91] Peter B Bosze S Horvath R. Biophysical characteristics of proteins and living cells exposed to the green tea polyphenol epigallocatechin-3-gallate (EGCg): review of recent advances from molecular mechanisms to nanomedicine and clinical trials. Eur Biophys J. (2017) 46:1–24. doi: 10.1007/s00249-016-1141-2, PMID: 27313063

[92] Wang G Wang J Momeni MR. Epigallocatechin-3-gallate and its nanoformulation in cervical cancer therapy: the role of genes, MicroRNA and DNA methylation patterns. Cancer Cell Int. (2023) 23:335. doi: 10.1186/s12935-023-03161-9, PMID: 38129839 PMC10740301

[93] Zhang J Cui H Yin J Wang Y Zhao Y Yu J. Separation and antioxidant activities of new acetylated EGCG compounds. Sci Rep. (2023) 13:20964. doi: 10.1038/s41598-023-48387-9, PMID: 38017306 PMC10684485

[94] Nian Y Zhang Y Ruan C Hu B. Update of the interaction between polyphenols and amyloid fibrils. Curr Opin Food Sci. (2022) 43:99–106. doi: 10.1016/j.cofs.2021.11.005

[95] Uddin MS Kabir MT Tewari D Mathew B Aleya L. Emerging signal regulating potential of small molecule biflavonoids to combat neuropathological insults of Alzheimer's disease. Sci Total Environ. (2020) 700:134836. doi: 10.1016/j.scitotenv.2019.134836, PMID: 31704512

[96] Debnath K Shekhar S Kumar V Jana NR Jana NR. Efficient inhibition of protein aggregation, disintegration of aggregates, and lowering of cytotoxicity by green tea polyphenol-based self-assembled polymer nanoparticles. ACS Appl Mater Interfaces. (2016) 8:20309–18. doi: 10.1021/acsami.6b06853, PMID: 27427935

[97] Zhang J Zhou X Yu Q Yang L Sun D Zhou Y. Epigallocatechin-3-gallate (EGCG)-stabilized selenium nanoparticles coated with Tet-1 peptide to reduce amyloid-beta aggregation and cytotoxicity. ACS Appl Mater Interfaces. (2014) 6:8475–87. doi: 10.1021/am501341u, PMID: 24758520

[98] Lin X Liu W Dong X Sun Y. Epigallocatechin gallate-derived carbonized polymer dots: a multifunctional scavenger targeting Alzheimer's beta-amyloid plaques. Acta Biomater. (2023) 157:524–37. doi: 10.1016/j.actbio.2022.11.063 36503076

[99] Ullah F Liang A Rangel A Gyengesi E Niedermayer G Munch G. High bioavailability curcumin: an anti-inflammatory and neurosupportive bioactive nutrient for neurodegenerative diseases characterized by chronic neuroinflammation. Arch Toxicol. (2017) 91:1623–34. doi: 10.1007/s00204-017-1939-4, PMID: 28204864

[100] Pal S Maity S Sardar S Parvej H Das N Chakraborty J. Curcumin inhibits the Al(iii) and Zn(ii) induced amyloid fibrillation of β-lactoglobulin in vitro. RSC Adv. (2016) 6:111299–307. doi: 10.1039/C6RA24570F

[101] Wang C Xu L Cheng F Wang H Jia L. Curcumin induces structural change and reduces the growth of amyloid-β fibrils: a QCM-D study. RSC Adv. (2015) 5:30197–205. doi: 10.1039/C5RA02314A

[102] Doytchinova I Atanasova M Salamanova E Ivanov S Dimitrov I. Curcumin inhibits the primary nucleation of amyloid-Beta peptide: a molecular dynamics study. Biomol Ther. (2020) 10:1323. doi: 10.3390/biom10091323, PMID: 32942739 PMC7563689

[103] Brahmkhatri VP Sharma N Sunanda P D’souza A Raghothama S Atreya HS. Curcumin nanoconjugate inhibits aggregation of N-terminal region (Aβ-16) of an amyloid beta peptide. New J Chem. (2018) 42:19881–92. doi: 10.1039/C8NJ03541E

[104] Mirzaie Z Ansari M Kordestani SS Rezaei MH Mozafari M. Preparation and characterization of curcumin-loaded polymeric nanomicelles to interference with amyloidogenesis through glycation method. Biotechnol Appl Biochem. (2019) 66:537–44. doi: 10.1002/bab.1751, PMID: 30993734

[105] Mahjoob M Stochaj U. Curcumin nanoformulations to combat aging-related diseases. Ageing Res Rev. (2021) 69:101364. doi: 10.1016/j.arr.2021.101364, PMID: 34000462

[106] Giacomeli R Izoton JC Dos Santos RB Boeira SP Jesse CR Haas SE. Neuroprotective effects of curcumin lipid-core nanocapsules in a model Alzheimer's disease induced by beta-amyloid 1-42 peptide in aged female mice. Brain Res. (2019) 1721:146325. doi: 10.1016/j.brainres.2019.146325, PMID: 31325424

[107] Di Pietro Fernandes C Santana LF Dos Santos JR Fernandes DS Hiane PA Pott A. Nutraceutical potential of grape (*Vitis vinifera* L.) seed oil in oxidative stress, inflammation, obesity and metabolic alterations. Molecules. (2023) 28:7811. doi: 10.3390/molecules28237811 38067541 PMC10708499

[108] Li Y Liang M Li T Qu Y Jiang Y Shi H. Green process for the preparation of resveratrol-containing high oleic acid peanut oil. Ultrason Sonochem. (2023) 100:106604. doi: 10.1016/j.ultsonch.2023.106604, PMID: 37852116 PMC10590997

[109] Ayaz M Mosa OF Nawaz A Hamdoon AAE Elkhalifa MEM Sadiq A. Neuroprotective potentials of Lead phytochemicals against Alzheimer's disease with focus on oxidative stress-mediated signaling pathways: pharmacokinetic challenges, target specificity, clinical trials and future perspectives. Phytomedicine. (2023) 124:155272. doi: 10.1016/j.phymed.2023.155272 38181530

[110] Wang L Gao M Chen J Yang Z Sun J Wang Z. Resveratrol ameliorates pressure overload-induced cardiac dysfunction and attenuates autophagy in rats. J Cardiovasc Pharmacol. (2015) 66:376–82. doi: 10.1097/FJC.0000000000000290, PMID: 26167810

[111] Neves AR Lucio M Martins S Lima JL Reis S. Novel resveratrol nanodelivery systems based on lipid nanoparticles to enhance its oral bioavailability. Int J Nanomedicine. (2013) 8:177–87. doi: 10.2147/IJN.S37840 23326193 PMC3544347

[112] Yang L Wang W Chen J Wang N Zheng G. A comparative study of resveratrol and resveratrol-functional selenium nanoparticles: inhibiting amyloid beta aggregation and reactive oxygen species formation properties. J Biomed Mater Res A. (2018) 106:3034–41. doi: 10.1002/jbm.a.36493 30295993

[113] Li C Wang N Zheng G Yang L. Oral Administration of Resveratrol-Selenium-Peptide Nanocomposites Alleviates Alzheimer's disease-like pathogenesis by inhibiting Abeta aggregation and regulating gut microbiota. ACS Appl Mater Interfaces. (2021) 13:46406–20. doi: 10.1021/acsami.1c14818, PMID: 34569225

[114] Yang L Wang Y Zheng G Li Z Mei J. Resveratrol-loaded selenium/chitosan nano-flowers alleviate glucolipid metabolism disorder-associated cognitive impairment in Alzheimer's disease. Int J Biol Macromol. (2023) 239:124316. doi: 10.1016/j.ijbiomac.2023.124316, PMID: 37004937

[115] Mazewski C Liang K Gonzalez De Mejia E. Comparison of the effect of chemical composition of anthocyanin-rich plant extracts on colon cancer cell proliferation and their potential mechanism of action using in vitro, in silico, and biochemical assays. Food Chem. (2018) 242:378–88. doi: 10.1016/j.foodchem.2017.09.086, PMID: 29037704

[116] Su Q Su W Xing S Tan M. Enhanced stability of anthocyanins by cyclodextrin-metal organic frameworks: encapsulation mechanism and application as protecting agent for grape preservation. Carbohydr Polym. (2024) 326:121645. doi: 10.1016/j.carbpol.2023.121645, PMID: 38142106

[117] Ali Shah S Ullah I Lee HY Kim MO. Anthocyanins protect against ethanol-induced neuronal apoptosis via GABAB1 receptors intracellular signaling in prenatal rat hippocampal neurons. Mol Neurobiol. (2013) 48:257–69. doi: 10.1007/s12035-013-8458-y, PMID: 23645118

[118] Rehman SU Shah SA Ali T Chung JI Kim MO. Anthocyanins reversed D-galactose-induced oxidative stress and neuroinflammation mediated cognitive impairment in adult rats. Mol Neurobiol. (2017) 54:255–71. doi: 10.1007/s12035-015-9604-5 26738855

[119] Amin FU Shah SA Badshah H Khan M Kim MO. Anthocyanins encapsulated by PLGA@PEG nanoparticles potentially improved its free radical scavenging capabilities via p38/JNK pathway against Abeta(1-42)-induced oxidative stress. J Nanobiotechnol. (2017) 15:12. doi: 10.1186/s12951-016-0227-4, PMID: 28173812 PMC5297201

[120] Kim MJ Rehman SU Amin FU Kim MO. Enhanced neuroprotection of anthocyanin-loaded PEG-gold nanoparticles against Abeta(1-42)-induced neuroinflammation and neurodegeneration via the NF-(K)B/JNK/GSK3beta signaling pathway. Nanomedicine. (2017) 13:2533–44. doi: 10.1016/j.nano.2017.06.022 28736294

[121] Bae CR Park YK Cha YS. Quercetin-rich onion peel extract suppresses adipogenesis by down-regulating adipogenic transcription factors and gene expression in 3T3-L1 adipocytes. J Sci Food Agric. (2014) 94:2655–60. doi: 10.1002/jsfa.6604, PMID: 24634340

[122] Bujor OC Ginies C Popa VI Dufour C. Phenolic compounds and antioxidant activity of lingonberry (*Vaccinium vitis-idaea* L.) leaf, stem and fruit at different harvest periods. Food Chem. (2018) 252:356–65. doi: 10.1016/j.foodchem.2018.01.052, PMID: 29478554

[123] Rajan VK Hasna CK Muraleedharan K. The natural food colorant Peonidin from cranberries as a potential radical scavenger – a DFT based mechanistic analysis. Food Chem. (2018) 262:184–90. doi: 10.1016/j.foodchem.2018.04.074, PMID: 29751907

[124] Wang T Yang C Li Z Li T Zhang R Zhao Y. Flavonoid 4,4′-dimethoxychalcone selectively eliminates senescent cells via activating ferritinophagy. Redox Biol. (2023) 69:103017. doi: 10.1016/j.redox.2023.103017 38176315 PMC10791569

[125] Zhang YN Zhu GH Liu W Chen XX Xie YY Xu JR. Discovery of the covalent SARS-CoV-2 M(pro) inhibitors from antiviral herbs via integrating target-based high-throughput screening and chemoproteomic approaches. J Med Virol. (2023) 95:e29208. doi: 10.1002/jmv.29208, PMID: 37947293

[126] Zhu J Cheng X Naumovski N Hu L Wang K. Epigenetic regulation by quercetin: a comprehensive review focused on its biological mechanisms. Crit Rev Food Sci Nutr. (2023) 7:1–20. doi: 10.1080/10408398.2023.2278760, PMID: 38062765

[127] Halevas E Mavroidi B Nday CM Tang J Smith GC Boukos N. Modified magnetic core-shell mesoporous silica nano-formulations with encapsulated quercetin exhibit anti-amyloid and antioxidant activity. J Inorg Biochem. (2020) 213:111271. doi: 10.1016/j.jinorgbio.2020.111271, PMID: 33069945

[128] Pinheiro RGR Granja A Loureiro JA Pereira MC Pinheiro M Neves AR. Quercetin lipid nanoparticles functionalized with transferrin for Alzheimer's disease. Eur J Pharm Sci. (2020) 148:105314. doi: 10.1016/j.ejps.2020.105314, PMID: 32200044

[129] Palmal S Maity AR Singh BK Basu S Jana NR Jana NR. Inhibition of amyloid fibril growth and dissolution of amyloid fibrils by curcumin-gold nanoparticles. Chemistry. (2014) 20:6184–91. doi: 10.1002/chem.201400079, PMID: 24691975

